# A Rare Presentation of Tuberculosis-Related Septic Shock

**DOI:** 10.7759/cureus.32528

**Published:** 2022-12-14

**Authors:** Louis Costanzo, Amara Shafi, Richard P Meier, Chetana Pendkar, David Smith

**Affiliations:** 1 Internal Medicine, State University of New York Downstate Medical Center, Brooklyn, USA; 2 Pulmonary and Critical Care Medicine, State University of New York Downstate Medical Center, Brooklyn, USA

**Keywords:** pulmonary tuberculosis, septic shock, sepsis, mycobacterium, multi-organ failure, malnutrition, bronchiectasis, acid fast

## Abstract

Septic shock with multi-organ dysfunction is an exceedingly rare, but known complication of untreated Mycobacterium tuberculosis (TB) infection. TB-associated cases of septic shock are predominantly reported in immunocompromised patients; however, it can manifest in a healthy individual if the infection is not treated. Through the interaction of lipoarabinomannan (LAM) on the mycobacterium cell wall with antigen-presenting cells, the bacteria may be able to survive in host cells for long periods of time. Without prompt treatment, TB may cause bronchiectasis and multi-organ failure. We report a case of a 24-year-old woman with untreated TB who developed widespread bronchiectasis and septic shock.

## Introduction

There are over 10 million newly diagnosed tuberculosis (TB) cases worldwide. More specifically, the incidence of TB in Guyana is currently greater than 100 per 100,000 [1]. TB-related cases of septic shock are remarkably rare and mainly seen in immunocompromised hosts. The following is a case of a malnourished female from Guyana who presented with severe malnutrition and septic shock in the setting of untreated Mycobacterium tuberculosis.

## Case presentation

A 24-year-old Guyanese woman with no past medical history presented to the emergency department with shortness of breath and weight loss over the past three months. She had also noted six weeks of a productive cough, hemoptysis, abdominal pain, and night sweats. The patient did not seek medical attention or undergo any previous treatment and remained home despite symptoms. Minimal history was obtained through a family member; the patient had not travelled recently, was not currently employed, and had a history of previously diagnosed TB which was unclear.

**Figure 1:**
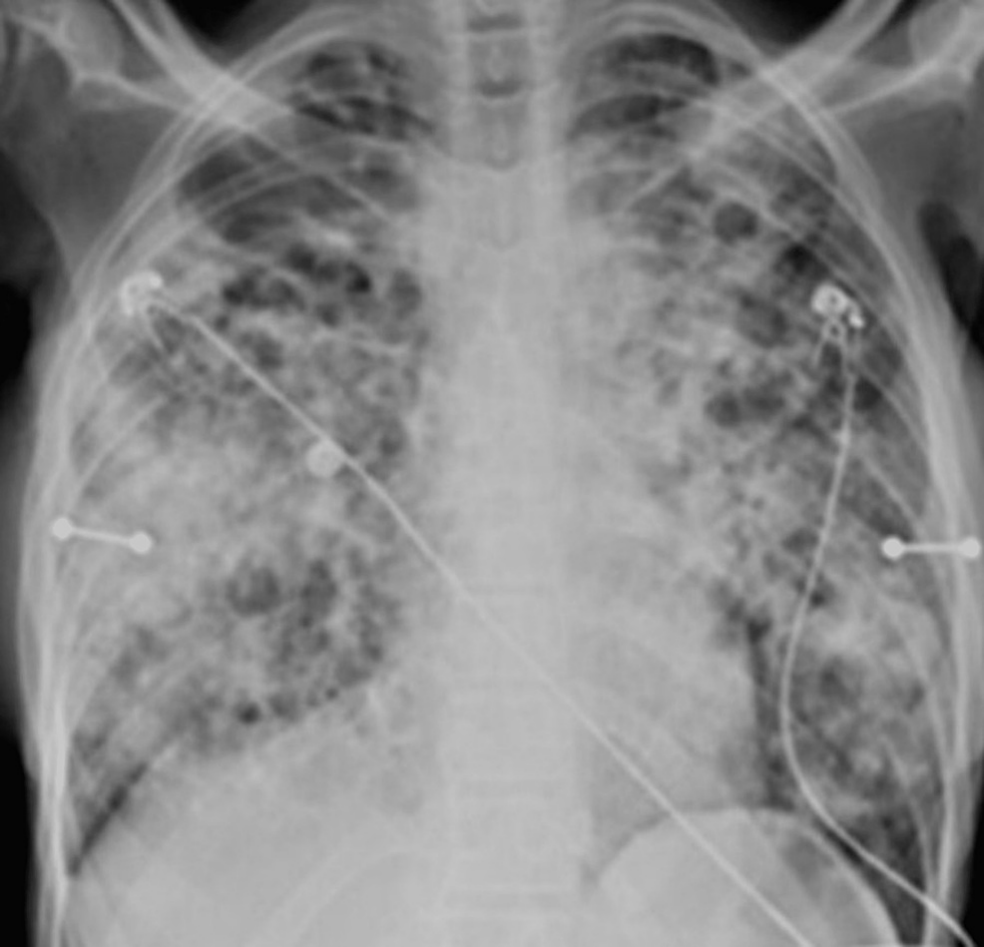
Initial chest X-ray A chest X-ray was taken in the emergency department with diffuse interstitial and airspace opacities bilaterally with interspersed lucencies.

In the emergency department, she was tachycardic to 140 bpm, hypotensive to 80/40 mmHg, tachypnic at 40 breaths per minute, and hypoxic to 82% on room air requiring a high-flow nasal cannula. She appeared ill and cachectic with a BMI of 10 kg/m2. The exam was further remarkable for accessory muscle use and diffuse coarse breath sounds in all lung fields. An ECG showed sinus tachycardia, lactate of 4 mmol/L, haemoglobin of 6.6 g/dL, and albumin of 2 g/dL. A chest X-ray was significant for diffuse cystic changes (Figure 1).

**Figure 2:**
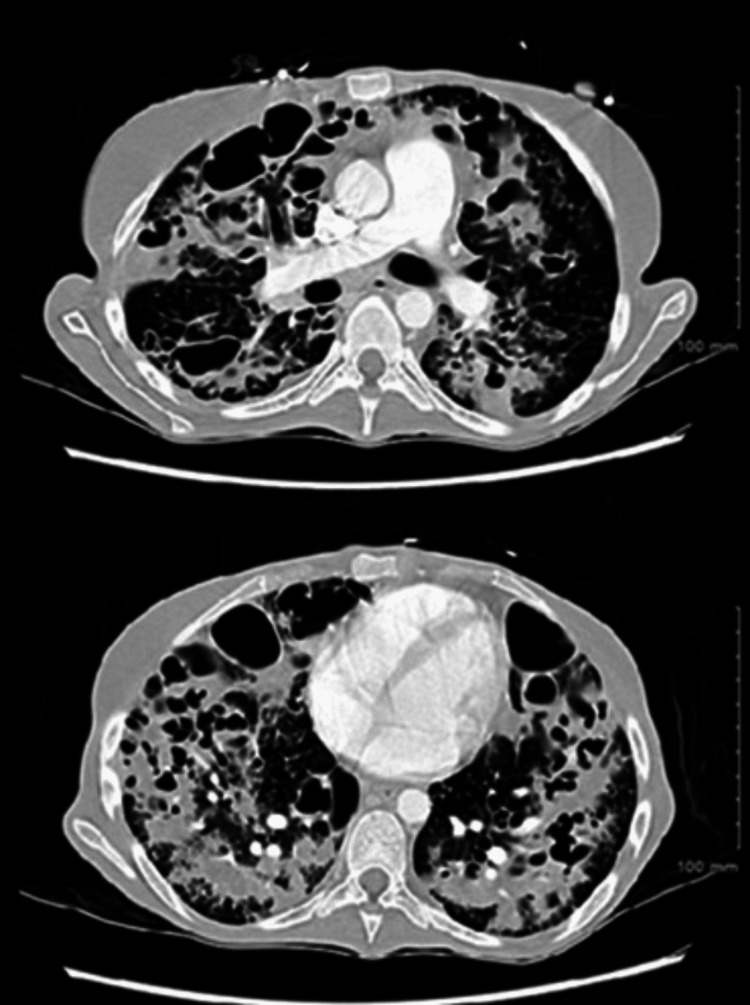
Varicose bronchiectasis CT scans of the chest with contrast showed extensive severe cystic and varicose bronchiectasis throughout the lung.

A CT chest scan demonstrated diffuse interstitial opacities and large cystic changes (Figure 2).

A right internal jugular central line was placed and she was given bolus fluids with minimal improvement in vital signs. She was started on broad-spectrum antibiotics and was admitted to the medical intensive care unit.

**Figure 3:**
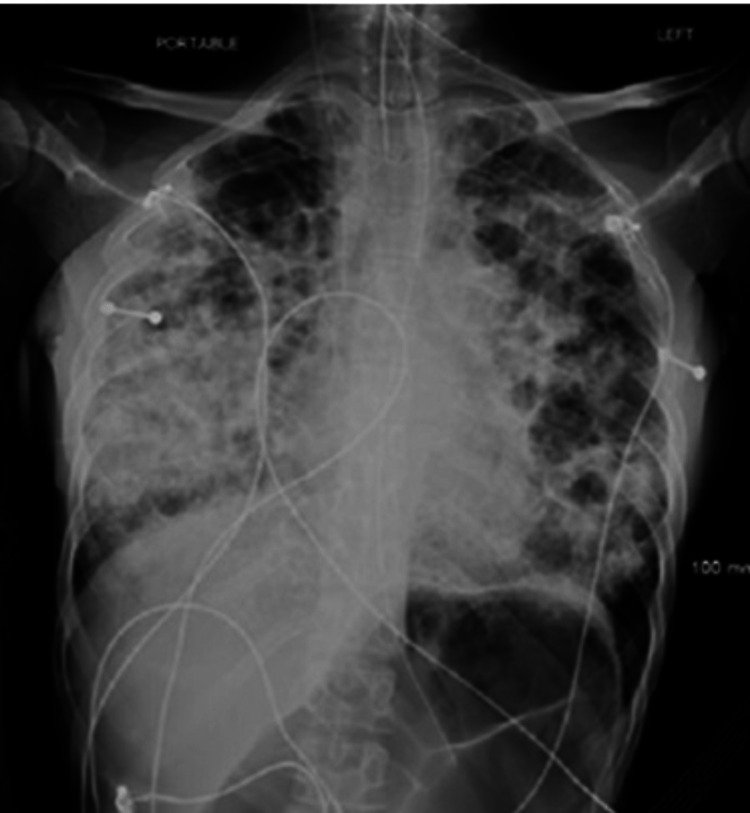
ICU chest X-ray with extensive cavitation and consolidation A chest X-ray was taken in the ICU redemonstrating cystic bronchiectasis and severe cavitary disease with extensive consolidations bilaterally.

In the ICU, standard blood cultures remained negative. Further workup including HIV testing and a respiratory viral panel was negative. Given the high suspicion of TB-related septic shock, the patient was started on empiric parenteral rifampin, isoniazid, pyrazinamide, and ethambutol (RIPE) therapy on hospital day 1 of 2 while waiting for acid-fast sputum culture results, which was positive the following day. She was also started on glucocorticoids due to TB-related adrenal insufficiency. A bedside echo was performed in the ICU which demonstrated collapse of the left ventricular walls at end-systole with a decreased left ventricular end-diastolic pressure, reduced cardiac output, a significantly collapsed inferior vena cava, and no signs of pericardial effusion. Overnight, the patient became severely hypoxic requiring intubation. Hours later she developed refractory hypotension despite four different vasopressors. A new chest X-ray redemonstrated bronchiectasis with extensive consolidations bilaterally (Figure 3).

She became pulseless shortly afterwards and advanced cardiac life support (ACLS) protocol was initiated. The patient remained in asystole throughout the arrest. Unfortunately, we were unable to achieve return of spontaneous circulation (ROSC) and the patient expired.

## Discussion

TB is a disease caused by Mycobacterium tuberculosis which is a unique bacterium with mycolic acid on the cell surface making it difficult to gram stain [2]. In a retrospective global cohort study, Kethireddy et al. were able to isolate TB as the causative agent of septic shock in only 1% of cases [3]. Most patients can recover from mild sepsis, but the mortality rate increases to 50% as they develop septic shock [4]. Mortality risk factors of patients with TB requiring ICU admission have been documented; however, there are no conventions in place to improve mortality of TB-related septic shock. Some of these risk factors that predict in-hospital mortality include acute respiratory failure requiring mechanical ventilation, acute respiratory distress syndrome (ARDS), co-infection with the HIV virus, extensive fibrocavitary disease and consolidation on chest radiographs, and multiple organ failure [5]. 

It is important to recognize that some cases of TB-related severe sepsis and septic shock will improve with early administration of parenteral anti-TB therapy. Arya et al. described a similar presentation of TB-related septic shock involving a patient with high-mortality risk factors which demonstrated the effectiveness of initiating empiric therapy based on clinical suspicion of TB [6]. It is the high index of suspicion in these cases that likely led to increased patient survival rates. A retrospective cohort study by Hazard et al. concluded that empiric anti-TB therapy was associated with improved survival rates in patients with severe sepsis [7].

Pathophysiology of TB-related shock

A TB infection results from inhalation of airborne particles that are <5μ in diameter, those of which carry few organisms. Upon entering the upper respiratory tract, the droplets deposit into the subpleural airspaces. Many particles are cleared by host alveolar macrophages; however, the TB bacilli can replicate within an activated host macrophage causing a primary infection.

**Figure 4:**
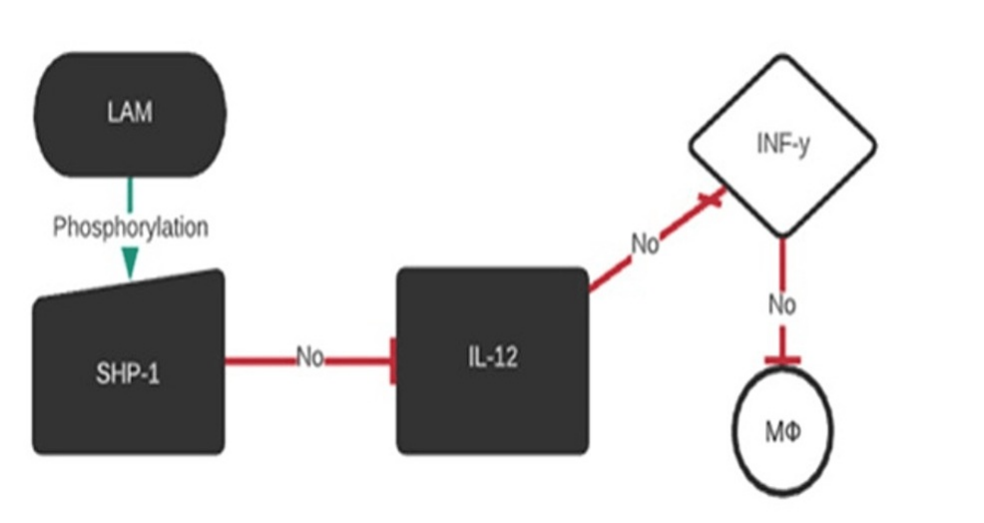
Survival of tuberculosis in host cells Proposed mechanism in which lipoarabinomannan (LAM) directly activates SHP-1 through phosphorylation. SHP-1, a signaling molecule that regulates many different cellular processes, may eventually deactivate proinflammatory cytokines such as IL-12, that plays a role in macrophage activation. This inability to release INF-y and activate macrophages promotes intracellular survival of TB in host cells [8,9].

Lipoarabinomannan (LAM), a glycolipid on the mycobacterium cell wall with virulent properties, interacts with carbohydrate recognition domains on the surfaces of macrophages and dendritic cells. By inhibiting SHP-1, a tyrosine-protein phosphatase, mycobacterium may be able to survive in host cells for long periods (Figure 4).

Without prompt treatment, TB can continue to spread bronchogenically and the caseous material causes the destruction of elastic and muscular components of bronchial walls resulting in widespread bronchiectasis, as seen in our patient. 

TB continues to spread both lymphatically and hematogenously causing a systemic response. Proinflammatory mediators promote leukocytosis and stimulate the release of other cytokines, all of which act as pyrogens, activate WBCs and macrophages, promote chemotaxis, cause immunosuppression, and stimulate both coagulation and fibrinolytic activation [10]. Arachidonic acid and adhesion molecules further cause vascular permeability and migration of aforementioned leukocytes. There is a link between tumour necrosis factor-α (TNF-α) and activation of the complement pathway, which further enhances neutrophil trafficking and inflammation [11]. This leads to tissue ischemia, cytopathic injury, and increased programmed cell death, eventually resulting in widespread multi-organ damage [12]. The rearrangement and distribution of intravascular fluid, inhibition of vasopressin release, and upregulation of vasoactive mediators such as nitric oxide results in hypotension or shock, as seen in our patient.

At the level of the lung, endothelial damage in the respiratory vasculature disrupts blood flow and increases microvascular penetrability, leading to interstitial and alveolar pulmonary edema [13]. The edematous fluid protein to plasma protein ratio is nearly 0.95, which is significantly higher than those with cardiogenic pulmonary edema [14]. This proteinaceous fluid destroys pneumocytes which increases surface tension, traps leukocytes, and further damages lung vasculature. Diffuse alveolar damage (DAD) ensues, a significant ventilation/perfusion mismatch occurs, and ARDS develops. When widespread TB-related lung damage occurs, ARDS is hardly reversible, especially when complicated by multi-organ failure.

TB and adrenal insufficiency

TB is one of the most common causes of adrenal insufficiency worldwide and a disseminated TB infection has the potential to spread to the adrenal glands. TB-related adrenal insufficiency may manifest as an acute adrenal crisis, especially in the setting of significant stress caused by a new infection. This is, in part, due to mycobacterial destruction and caseous necrosis of the adrenocortical tissue [15]. We considered our patient’s symptoms to be a manifestation of a TB-related adrenal crisis; however, it is uncommon for the adrenal glands to be the only infected organ. Adrenal involvement was found in 6% of patients with active TB in a 28-year-autopsy series, and only one-quarter of these cases listed the adrenals as the only site of infection [16]. Nonetheless, adrenal insufficiency is always important to consider in cases involving disseminated TB. To aid in the diagnosis, one can consider sending a serum cortisol level, adrenocorticotropic hormone (ACTH) stimulation testing, and utilizing an abdominal CT to evaluate for hemorrhage, inflammatory cell infiltration, or adrenal enlargement. 

Treatment of TB-related septic shock

Immediate management involves the timely administration of supplemental oxygen and fluids [17]. The administration of balanced crystalloid IV fluids like ringers lactate should be started within the first hour. Two empiric antibiotics intravenous antibiotics from different classes should also be administered to cover for the most common pathogens. 

Next, an acid-fast sputum smear and culture should be collected, and laboratory data including complete blood count (CBC), complete metabolic panel (CMP), prothrombin and partial thromboplastin time (PT/PTT), D-dimer, serum lactate, blood cultures, arterial blood gas (ABG), HIV Ag/Ab, beta-human chorionic gonadotropin (B-hCG), and procalcitonin should be obtained. Clinicians should consider starting empiric TB treatment while awaiting acid-fast bacilli (AFB) smear results. If the clinical presentation and imaging findings are consistent with TB and the patient is in septic shock related to TB, anti-TB medications need to be administered as soon as possible. It is important that clinicians do not wait for the sputum results before initiating treatment. With severe sepsis and septic shock, the timing of antibiotics directly affects the outcome. This is also valid for a tuberculous-related septic shock [3]. Further, clinicians must consider a parenteral route for treatment in patients with suspected disseminated TB and septic shock. This is because oral absorption is unpredictable considering poor splanchnic circulation in a state of shock and poor absorption may lead to poor outcomes.

The preferred regimen for the treatment of pulmonary and extrapulmonary manifestations of TB including TB-related sepsis shock without HIV includes two months of RIPE, followed by a continuation phase for an additional four months [18].

Vasopressors are useful in patients who are continuously hypotensive despite adequate fluid management. First-line vasopressors include norepinephrine; second or third agents such as epinephrine, dobutamine, or vasopressin may be used in the setting of reduced cardiac output. Steroids can be considered for six weeks in those with TB meningitis or TB pericarditis, in those with persistent septic shock despite adequate fluid resuscitation, and in those when TB adrenalitis is suspected.

## Conclusions

This case highlights the possibility of TB-related septic shock in the critical care setting and the importance of prompt intervention, including treatment with anti-TB agents and therapies to correct hypoxemia and hemodynamic instability. Clinicians should consider administering parenteral therapy as soon as possible while waiting for additional results, which may likely contribute to positive patient outcomes. This case also calls attention to possible mechanisms contributing to TB-related septic shock, as well as acknowledges the link between disseminated TB and adrenal insufficiency.
